# Richard Hynes, Erkki Ruoslahti, and Timothy Springer receive Lasker prize for pioneering work on integrins

**DOI:** 10.1172/JCI164374

**Published:** 2022-09-28

**Authors:** Hidayatullah G. Munshi

Integrins are transmembrane glycoprotein receptors with central roles in cell-cell and cell–extracellular matrix (ECM) interactions (1, 2). These interactions are paramount to many biological processes, from early embryonic development to mature tissue function (1, 2). Integrin signaling also regulates various pathological processes, with critical roles in thrombotic disease, inflammation, and cancer (1–3). Thus, there has been a long-standing interest in targeting integrins to treat human disease (4). Accordingly, several potent and highly specific inhibitors targeting integrins have entered clinical phase testing, some earning Food and Drug Administration (FDA) approval. For example, inhibitors targeting the αIIbβ3 integrin (also known as glycoprotein IIb/IIIa) have been approved for acute coronary syndrome and thrombotic cardiovascular events (4). Similarly, antibodies targeting the leukocyte integrins α_4_β_7_ and α_4_β_1_ have been approved for inflammatory bowel disease and multiple sclerosis (4).

Given the profound impact of integrins on basic science research and clinical practice, the Lasker Foundation recognizes Richard Hynes (Massachusetts Institute of Technology), Erkki Ruoslahti (Sanford Burnham Prebys Medical Discovery Institute), and Timothy Springer (Boston Children’s Hospital/Harvard Medical School) with the 2022 Albert Lasker Basic Medical Research Award for discoveries related to integrins. Specifically, Hynes and Ruoslahti were instrumental in identifying the role of the ECM molecule fibronectin in adhesion and discovering the “fibronectin receptor,” now part of the class of receptors known as integrins. In a parallel series of studies, Springer made foundational contributions to the discovery of select leukocyte integrins and uncovering their many roles in leukocyte biology.

## The winding road to fibronectin

The research leading to the discovery of integrins dates to the early 1970s. Researchers noted that viral-transformed cells grew much faster than nontransformed cells. Intending to find an integral membrane protein involved in growth control, Hynes explored the molecular differences between the surfaces of nonmalignant and transformed tumor cells (5). He discovered that transformed cells had diminished expression of a large external protein, which he referred to as the LETS (large external transformation sensitive) protein (5, 6). His team went on to show that adding the purified LETS protein to transformed cells lacking the protein significantly impacted cell adhesion, morphology, and migration (5). They also showed that the LETS protein affected the cytoskeleton, with actin fibrils coaligning across the cell surface (7). Thus, they concluded that they had identified an ECM protein rather than an integral protein.

Around the same time, Ruoslahti and his team were also conducting studies to isolate cell-surface proteins that might be altered between normal and cancer cells. Given the invasive nature of cancer cells, they hypothesized that there must be differences in cell-surface proteins between cancer cells and normal cells (8). They focused their efforts on identifying differentially expressed proteins that mediate cell recognition to guide cell movement and positioning (8). Using antibodies generated against proteins released from the surface of embryonal fibroblasts, they identified a protein absent in viral-transformed fibroblasts (8, 9). Like Hynes and his team, Ruoslahti and colleagues showed that their protein, initially named SF protein for “surface of fibroblasts,” mediated cell attachment. After sequencing a fragment of the SF protein and through studies using synthetic peptides, they found that the Arg-Gly-Asp (RGD) sequence was the minimal cell-binding sequence in their SF protein (10).

Subsequent studies demonstrated that the SF protein and the LETS protein were, in fact, the same protein (5, 8). Ruoslahti and his team later renamed the protein fibronectin (8). Interestingly, the plasma form of fibronectin was identical to a protein previously known as the “cold insoluble globulin” (8), raising important questions regarding the role of fibronectin in both physiologic and pathologic processes. Additional studies by Hynes, Ruoslahti, and others further explored the roles of fibronectin, importantly demonstrating that RGD peptides may be useful in blocking adhesion-dependent biological processes, such as platelet aggregation.

## The hunt for a fibronectin receptor

Following the identification of fibronectin, the search was underway for a potential “fibronectin receptor.” The Ruoslahti group took advantage of synthetic peptides based on the RGD-binding site in fibronectin to isolate the fibronectin receptor. Initially, instead of identifying the fibronectin receptor, they isolated the receptor for vitronectin and subsequently isolated the receptor for fibronectin (11, 12). Based on peptide sequences, they determined that fibronectin and vitronectin receptors were heterodimeric proteins (11, 12). While the two receptors recognized different proteins, in both instances, recognition depended on the RGD sequence of the ECM proteins (8).

The Hynes group also worked to identify the fibronectin receptor. Expanding on previous studies showing that fibronectin can bind platelets and promote their spreading, they hypothesized that the GPIIb/IIIa receptor on platelets might, in fact, be a fibronectin receptor (5). By crosslinking the cell-binding fragment of fibronectin to an inducible receptor on platelets, they identified the receptor to be GPIIb/IIIa and showed that the GPIIb/IIIa receptor binding to fibronectin was RGD dependent (13). Subsequently, the Hynes group successfully cloned and sequenced one of the subunits of the fibronectin receptor, discovering it to be a transmembrane protein (14). They named the protein “integrin” for the integral membrane protein linking the ECM to the cytoskeleton (14).

## The surprising discovery of leukocyte integrins

While the Hynes and Ruoslahti groups were focused on identifying ECM receptors on the surface of epithelial cells, fibroblasts, and platelets, the Springer group was busy discovering and characterizing the role of receptors on leukocytes. Having gained expertise in antibody generation during his postdoctoral training with Cesar Milstein, Springer used antibodies to identify Mac-1 as a marker for myeloid cells (15). Then, using function-blocking monoclonal antibodies, Springer went on to identify proteins that play critical roles in the killing of tumor cells by cytotoxic T lymphocytes. He discovered LFA-1 (lymphocyte function-associated antigen-1) on cytotoxic T cells and showed that antibodies targeting LFA-1 prevent T cell conjugate formation with the target cells, thereby impeding cell-mediated cytotoxicity (16). The Springer group went on to show that both LFA-1 and Mac-1 form heterodimers, with an identical β subunit but unique α subunits (17). Using monoclonal antibodies against the β subunit, the Springer group also identified a third heterodimeric protein, p150,95, sharing the common β subunit (15). When the Springer lab sequenced the cDNA of the β subunit of LFA-1 and Mac-1, they found significant homology to the β subunit of the fibronectin receptor (18). Moreover, the N-terminal sequences of the α subunits of the vitronectin receptor and the platelet glycoprotein IIb/IIIa protein were found to be homologous to the α subunits of LFA-1 and Mac-1 (15). These unexpected results suggested that LFA-1, Mac-1, and the p150,95 proteins were related to the ECM receptors characterized by Hynes, Ruoslahti, and others. These proteins were later shown to belong to the integrin superfamily of proteins (15).

Subsequently, the α subunits of LFA-1, Mac-1, and p150,95 were designated as CD11a, CD11b, and CD11c, respectively, while the β subunit was designated as CD18. The expression of all three leukocyte integrins is restricted to immune cells, with LFA-1 expression seen in virtually all immune cells (15). This work has been directly related to human disease, as defects in the common β subunit cause leukocytes to lack cell-surface expression of all 3 leukocyte integrins, resulting in leukocyte adhesion deficiency (LAD) (19). LAD is a primary immunodeficiency stemming from a defect in the ability of leukocytes to properly migrate to sites of infection. Given their inability to kill offending microbes, patients with LAD develop recurrent life-threatening bacterial infections (19).

## Integrin research continues to excite and deliver

Following these seminal discoveries, all three investigators continued their research on integrins and their role in cell-cell and cell-ECM interactions. Hynes and his team cloned and characterized several additional cell adhesion molecules, both ECM proteins and other proteins involved in cell-cell and cell-ECM interactions. They generated transgenic mice lacking adhesion molecules and used them to understand the role of adhesion molecules in normal and pathological processes, including inflammation and cancer. In recent years, Hynes has focused his research on exploring the role of ECM proteins in cancer progression, particularly the intersection between ECM and metastasis. To that end, his laboratory has developed nanobodies directed against ECM proteins and successfully used them to image both primary tumors and metastases (20). They envision that selective targeting of tumor ECMs may enable the effective delivery of therapeutics to tumors.

Ruoslahti has also continued his work to define how metastatic cells use adhesion molecules to home to appropriate sites in the body. His team has since introduced the concept of “vascular ZIP codes” based on the molecular differences in the blood vessels of various normal tissue as a possible explanation (21). His work demonstrated that blood vessels in tumors differ from normal vessels, in part by the differential expression of specific integrins, thus offering a potential explanation for the apparent organotropism of select tumor types. Consequently, he showed that peptides could be used to target and deliver drugs and nanoparticles to tumors. Additionally, they have discovered that, by activating the CendR transcytosis pathway, some of these peptides can penetrate deep into the tumors, offering a novel strategy for drug delivery (21). Their prototype tumor-penetrating peptide iRGD, which recognizes the α_v_β_3_/α_v_β_5_ integrin, is in phase 2 trials for pancreatic and other gastrointestinal malignancies (21).

The Springer group also continued their research on leukocyte cell–adhesion molecules. Their discovery of LFA-1 and other leukocyte cell–adhesion proteins led to the development and subsequent FDA approval of antibodies and other biologics to treat psoriasis (22). His group also discovered the process of leukocyte diapedesis, including the crucial role of integrins in mediating the adhesion of leukocytes to vascular endothelium and subsequent migration through the vessel wall. This work led to the development of an antibody to integrin α_4_β_7_, a lymphocyte homing receptor for mucosal tissues. Based on the observation that targeting α_4_β_7_ can rapidly resolve colitis, an antibody targeting α_4_β_7_ also entered clinical testing, earning FDA approval for severe inflammatory bowel diseases (4). In additional studies, Springer and his group have contributed to our understanding of how conformational changes and tensile forces activate integrins.

As demonstrated by their work, Hynes, Ruoslahti, and Springer have revolutionized basic and translational research. They have clearly shown that integrins and other cell adhesion molecules impact every arena of medical research, offering unprecedented insight into previously poorly understood cellular processes. Their work has enabled the successful development of therapies that have helped countless patients with cardiovascular and inflammatory diseases. Their discoveries also paved the way for successful imaging and delivery of therapeutics to tumors. The recipients of this year’s Lasker Award have laid the groundwork for further understanding the role of integrins in normal and pathological processes and for developing additional novel therapies targeting cell-cell and cell-ECM interactions.

## Version 1 09/28/2022

Electronic publication

## Version 2 10/03/2022

Electronic publication
